# A247 ACTIVATION OF VISCERAL AFFERENT NEURONS INVOLVED IN NOCICEPTION BY A BACTERIUM ASSOCIATED WITH INFLAMMATORY BOWEL DISEASE

**DOI:** 10.1093/jcag/gwae059.247

**Published:** 2025-02-10

**Authors:** R Nakhle, H M Wood, C C Baker, D E Reed, A E Lomax

**Affiliations:** Queen’s University, Kingston, ON, Canada; Queen’s University, Kingston, ON, Canada; Queen’s University, Kingston, ON, Canada; Queen’s University, Kingston, ON, Canada; Queen’s University, Kingston, ON, Canada

## Abstract

**Background:**

Inflammatory bowel disease (IBD) is characterized by chronic relapsing inflammation of the gastrointestinal tract. Severe abdominal pain is a common symptom of IBD, yet effective pain treatments are limited. Proteolytic activity is elevated in IBD compared to healthy individuals. Recently, protease production by the gut bacterium *Bacteroides vulgatus* has been correlated with the severity of IBD.

**Aims:**

Since proteases acting on protease-activated receptor 2 (PAR-2) can activate visceral pain pathways, we test the hypothesis that mediators from *B. vulgatus* activate visceral pain pathways and can directly activate dorsal root ganglia (DRG) neurons, altering pain signalling.

**Methods:**

The effects of *B. vulgatus* ATCC 8482 supernatant or brain heart infusion (BHI) growth media on nociceptive neurons were assessed using *ex-vivo* single-unit afferent nerve recordings from C57bl/6 mouse colons. To further examine cellular mechanisms, Ca^2+^ imaging was performed on dissociated DRG neurons following the superfusion of *B. vulgatus* culture supernatant. As TRPV1 is an ion channel expressed mainly on nociceptive neurons, the effect of *B vulgatus* culture supernatant on Ca^2+^ influx in response to the TRPV1 agonist capsaicin was also quantified. “N” represents mice, while “n” represents single afferent units or DRG neurons.

**Results:**

Perfusion of *B. vulgatus* supernatant (1:20) through the lumen of murine colons significantly increased basal firing frequency by 50% (p < 0.001, N = 8, n = 38) and mechanosensitivity of colonic afferent axons by 48% (p < 0.05, N = 8). Interestingly, only the putative nociceptor high-threshold units were activated upon perfusion of this bacterial supernatant (p < 0.01, N = 8, n = 13), while the wide-dynamic range units were unaffected (p > 0.05, N = 8, n = 30). The BHI growth media did not alter basal activity (p > 0.05, N = 6, n = 23) or afferent nerve mechanosensitivity (p > 0.05, N = 6). Superfusion of *B. vulgatus* supernatant (1:100) increased intracellular [Ca^2+^] by 16% (p < 0.0001, N= 7, n = 51). Furthermore, exposure to the *B. vulgatus* supernatant significantly elevated Ca^2+^ influx in response to the TRPV1 agonist capsaicin (100 nM; p < 0.0001, n = 77, N = 7), while the BHI media did not alter intracellular [Ca^2+^] in response to capsaicin (p > 0.05, n = 66, N = 7).

**Conclusions:**

Mediators released by *Bacteroides vulgatus* can activate visceral afferent nerves involved in nociception. Additionally, *B. vulgatus* can directly modulate DRG neuronal Ca^2+^ homeostasis and sensitize TRPV1 in these neurons. Future studies will explore whether proteases are responsible.

**Funding Agencies:**

CIHR

